# Changes in Lung Ultrasound in Systemic Sclerosis Before and After Rehabilitation: A Comparative Study Between People with and Without Interstitial Lung Disease

**DOI:** 10.3390/arm93030009

**Published:** 2025-05-20

**Authors:** Samantha Gomes de Alegria, Matheus Mello da Silva, Jéssica Gabriela Messias Oliveira, Beatriz Luiza Pinheiro Alves de Azevedo, Nathália Alves de Oliveira Saraiva, Isabelle da Nóbrega Ferreira, Joana Acar Silva, Thiago Thomaz Mafort, Cláudia Henrique da Costa, Agnaldo José Lopes

**Affiliations:** 1 Post-Graduation Programme in Medical Sciences, School of Medical Sciences, Universidade do Estado do Rio de Janeiro (UERJ), Avenida Professor Manoel de Abreu, 444, 2º andar, Vila Isabel, Rio de Janeiro 20550-170, Braziltmafort@gmail.com (T.T.M.); agnaldolopes.uerj@gmail.com (A.J.L.); 2 Rehabilitation Sciences Post-Graduation Programme, Centro Universitário Augusto Motta (UNISUAM), Rua Dona Isabel, 94, Bonsucesso, Rio de Janeiro 21032-060, Brazil; mathhews.melo@gmail.com (M.M.d.S.);; 3 Department of Pulmonology, Pedro Ernesto University Hospital, Boulevard 28 de Setembro, 77, Vila Isabel, Rio de Janeiro 20551-030, Brazil

**Keywords:** systemic sclerosis, rehabilitation program, lung ultrasound

## Abstract

**Highlights:**

**What are the main findings?**

- Lung ultrasound can be a valuable tool for evaluating women with systemic sclerosis before and after rehabilitation programs.
- A physiotherapist-guided rehabilitation program can serve as a non-pharmacological treatment strategy for women with systemic sclerosis.

**What is the implication of the main finding?**

- Our data support the inclusion of lung ultrasound in the routine monitoring of women with systemic sclerosis and using a physiotherapy-guided rehabilitation program, as well as the use of physiotherapy-guided rehabilitation programs as non-pharmacological treatments for this population.
- Further evaluation of the use of lung ultrasound and the benefits of physiotherapy-guided rehabilitation programs requires more clinical trials with larger sample sizes of people with systemic sclerosis.

**Abstract:**

There is limited research on the impact of a physiotherapy-guided rehabilitation program (PGRP) on lung structure in systemic sclerosis (SSc). Lung ultrasound (LUS) has been used for over a decade to identify interstitial lung disease associated with SSc (SSc-ILD). This study aims to evaluate the impact of a PGRP on LUS signals in women with SSc-ILD and women without ILD (SSc-wILD). This is a longitudinal quasi-experimental study that included 33 women with SSc. The results show that changes in LUS were observed in 22 participants, especially B-lines > two. Before the PGRP the median of B-lines > two was three (0–7), while after the PGRP it was three (0–6) (*p* = 0.020). The aeration score was eight (0–16.5) pre-PGRP and three (0–16) post-PGRP (*p* = 0.013). Analyzing the impact of the PGRP on LUS signals in SSc-ILD and SSc-wILD groups, the main change observed was a reduction in B-lines > two between pre-PGRP and post-PGRP in the SSc-ILD group (*p* = 0.0004). The SSc-ILD group had a higher aeration score than the SSc-wILD group both pre-PGRP (*p* < 0.0001) and post-PGRP (*p* = 0.0001)]. In conclusion, LUS may be a complementary tool for assessing pre- and post-PGRP changes in people with SSc-ILD and SSc-wILD. Our data also suggest that the PGRP can elicit measurable changes in LUS findings in SSc, particularly in SSc-ILD. These findings support the inclusion of LUS in the routine monitoring of SSc and the use of a PGRP as a non-pharmacologic adjunctive strategy in SSc.

## 1. Introduction

Systemic sclerosis (SSc) is a rare and complex autoimmune disease of poorly understood etiology that affects connective tissue and is more common in women [1]. The pathophysiology is characterized by small-vessel vasculopathy, autoantibody production, and fibroblast dysfunction, leading to excessive deposition of extracellular matrix. Early signs of the disease include Raynaud’s phenomenon (RP) and fatigue [1]. Clinical manifestations and prognosis are variable, with most patients presenting with skin thickening and varying degrees of internal organ involvement [2]. People with SSc can be classified into two main clinical subtypes with different forms of disease progression, which are the diffuse cutaneous form and the limited cutaneous form. Individuals with diffuse cutaneous SSc present with rapid progression of skin thickening located proximal to the elbows or knees, with a higher risk of visceral involvement and higher mortality. On the other hand, in individuals with limited cutaneous SSc, skin thickening is restricted to areas distal to the elbows and knees, with or without facial involvement; these patients have less frequent visceral involvement, but they are at greater risk of developing advanced-stage pulmonary arterial hypertension [3].

Interstitial lung disease (ILD) associated with SSc is characterized by a diffuse infiltrative process in the lung parenchyma that can lead to tissue fibrosis and loss of lung function, and is a major cause of death and loss of health-related quality of life (HRQoL) in this population. Therefore, it is important to understand how this disease manifests in the respiratory system in order to better manage patients [4]. Accurate clinical, imaging, and laboratory tests are needed to phenotype people with SSc. Diagnosis of ILD is traditionally based on a chest computed tomography (CT) scan and reduction in forced vital capacity (FVC) and diffusing capacity of the lungs for carbon monoxide (DLco) on pulmonary function tests (PFTs). Nonspecific interstitial pneumonia in SSc-ILD is characterized by ground-glass opacities on CT. Reticulations and traction bronchiectasis are other possible findings that define a fibrotic phenotype. Usual interstitial pneumonia (UIP), defined by honeycombing, is less common in SSc-ILD. Lung ultrasound (LUS) has been used for over a decade to detect SSc-ILD and is currently undergoing standardization. Quantitative and qualitative analysis of B-lines and pleural line changes have been sought to detect ILD changes. LUS has the advantages of being radiation-free, rapid, low cost, and accessible in an outpatient setting, and has demonstrated good diagnostic accuracy for detecting ILD compared with the current gold standard of CT [5].

In recent years, there have been significant advances in the diagnostic criteria, clinical monitoring, and pharmacological management of people with SSc, leading to a better prognosis. Pulmonary involvement is usually progressive pulmonary fibrosis, the most common symptom of which is dyspnea on exertion. For decades, studies have focused on explanations in terms of deranged physiology based on pulmonary function data and altered anatomy based on chest CT findings [6]. Following a systematic review of the literature, the current recommendations of the European Alliance of Associations for Rheumatology (EULAR) for non-pharmacological management of SSc are based on physical exercise, patient education, and support for self-management of the disease, with the goal of improving hand function, orofacial mobility, HRQoL, exercise capacity, and ability to perform activities of daily living (ADLs), with maintenance of these improvements up to 9 weeks after treatment initiation [7]. However, there is a gap in the literature regarding the effect of a physiotherapy-guided rehabilitation program (PGRP) on lung structure.

There are relatively few studies on the response of the lungs to an exercise program in people with SSc. Although physical exercise has been shown to be safe and beneficial in improving physical capacity, vascular function, and HRQoL in people with SSc, knowledge of its effects on the respiratory system is limited. Therefore, the present study aims to evaluate the impact of a PGRP on LUS signals in women with SSc-ILD and women without ILD (SSc-wILD).

## 2. Materials and Methods

Between November 2021 and March 2024, a longitudinal quasi-experimental study was conducted that included 33 women meeting the diagnostic criteria for SSc [2], with ILD associated or not, aged ≥18 years, recruited at the Pedro Ernesto University Hospital of the State University of Rio de Janeiro, Rio de Janeiro, Brazil. A chest CT scan performed up to 3 months prior to study enrollment was used to diagnose ILD [8]. The following exclusion criteria were applied: presence of severe comorbidities that caused serious difficulties in performing physical therapy activities; history of orthopedic surgery involving the spine and upper/lower limbs requiring an assistive device for locomotion; participation in any rehabilitation protocol in the last 6 months; and inability to perform the tests or the rehabilitation program supervised by a physical therapist. No changes in antirheumatic pharmacological treatment were made during the PRGP period. All participants signed the informed consent form, and the protocol was approved by the Research Ethics Committee of the Pedro Ernesto University Hospital of the State University of Rio de Janeiro, Rio de Janeiro, Brazil, under number CAAE-52759521.2.0000.5259. The trial was registered on ClinicalTrials.gov under the number NCT05041868.

**Figure 1:**
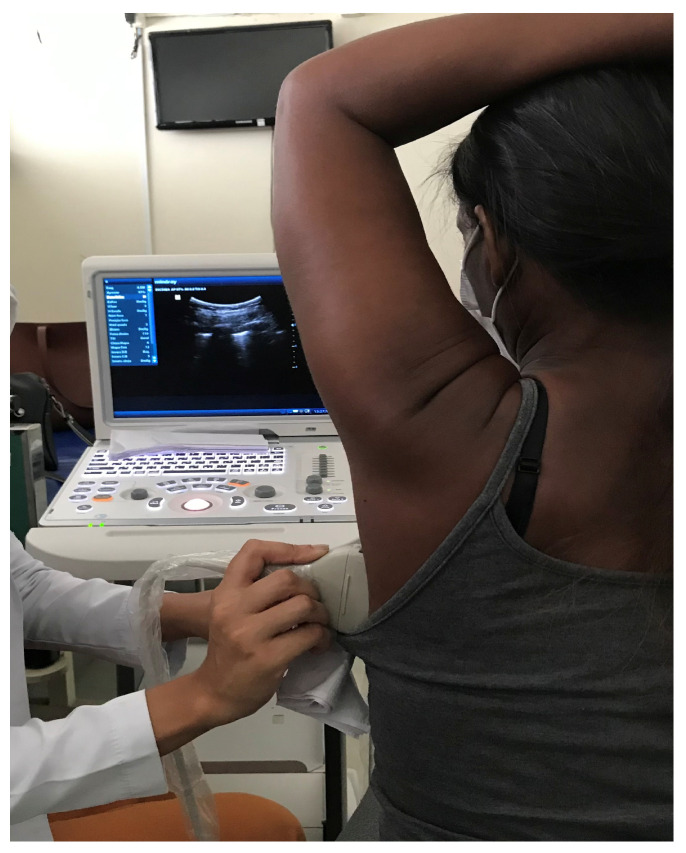
Acquisition of lung ultrasound images in a patient diagnosed with SSc-ILD. Source: author.

LUS images were collected on the day of assessment for enrollment in the PGRP and on the day of reassessment after 12 weeks of participation (Figure 1). The device (Mobile Trolley UMT-150, Mindray, Shenzhen, China) was used with a 7.5–10 MHz multifrequency linear transducer or a 3.5–5 MHz convex transducer in B mode. The examinations were performed by a team of six clinicians experienced in the method, with each examination being performed by two physicians, who reached a consensus in case of disagreement. Ultrasound examinations were performed in six areas of each hemithorax (two anterior, two lateral, and two posterior). The LUS images were analyzed to identify the following signs: B-lines > two, coalescent B-lines, and subpleural consolidations. To classify the degree of lung aeration by LUS, we used the aeration score, which is based on the ultrasound patterns observed by the clinicians in the 12 different areas of the chest. Each individually assessed area is assigned a score from zero to three, depending on the degree of loss of lung aeration observed. A score of zero is given if there are A-lines or fewer than two isolated B-lines, which correspond to preserved lung aeration. A score of one is assigned if three or more well-defined and separate B-lines are visualized, indicating mild loss of aeration. A score of two is assigned if the B-lines coalesce, which represents a moderate loss of aeration. Finally, a score of three is assigned if there is visible lung consolidation, which represents a severe loss of aeration in the region being evaluated. The sum of all 12 areas evaluated by LUS represents the aeration score (0 to 36 points) [9,10].

The participants underwent a PGRP, performed three times a week for 12 weeks. After the assessment, the participants received a prescription for the PGRP exercises through a guide with illustrations and descriptions of all the exercises, in addition to a demonstration video to improve accessibility. On the same day, participants practiced the exercises in the presence of a physical therapist. Each session included muscle strengthening, aerobic resistance, and flexibility exercises, and lasted 60 min. The session began with 5 min of warm-up exercises, followed by 20 min of muscle strengthening of large groups (flexion, extension, adduction, abduction, and rotations) and resistance exercises using light weights and functional movements of the upper and lower limbs, including open and closed kinetic chain exercises. Subsequently, 10 min of postural control training through proprioceptive exercises on the ground were performed, followed by 20 min of aerobic training in functional circuits. Finally, 5 min of stretching and relaxation using calisthenic exercises were performed. Participants were contacted weekly by telephone by a physical therapist who monitored the progress of the treatment. Participants were reassessed 12 weeks after inclusion in the protocol, and the PGRP program was then terminated [11,12,13].

Data analysis was performed using IBM SPSS Statistics version 26 software (IBM Corp., Armonk, NY, USA). Normality of variables was assessed by the Shapiro–Wilk test, and results were expressed by measures of central tendency and adequate dispersion for numerical data, and frequency and percentage for categorical data. Comparison between pre- and post-PGRP measurements was assessed by the Wilcoxon signed-rank test for numerical data and by the chi-square (χ^2^) test, Fisher’s exact test, or one-sided McNemar exact test for categorical data. Comparison between SSc-ILD and SSc-wILD with the numerical variables of the LUS was analyzed by the Mann–Whitney test, and the Fisher’s exact test was used for categorical variables. The absolute delta was obtained by subtracting the post-PGRP value from the pre-PGRP value. Statistical significance was considered if *p* < 0.05.

## 3. Results

### 3.1. Study Population

Of the 42 women diagnosed with SSc who met the inclusion criteria for participation in the PGRP, 33 completed the program. There were three exclusions due to difficulty in locomotion and six exclusions due to treatment abandonment. The mean age was 48.8 ± 13 years. The median time since diagnosis was 8 (3–15) years. On chest CT scan, 18 (54.5%) participants were diagnosed with ILD. The individual characteristics, anthropometric measurements, clinical characteristics, and serology for SSc marker antibodies at baseline are shown in Table 1.

### 3.2. Lung Ultrasound—Number of Patients

Changes in LUS signals were observed in the examination of 22 (66.7%) participants both pre-PGRP and post-PGRP. The main change observed was B-lines > two present in 21 (63.6%) participants both pre-PGRP and post-PGRP. There were no changes in the frequencies of LUS signals for coalescent B-lines (*n* = 12, 36.4%) before and after rehabilitation. The frequency of subpleural consolidations was higher before rehabilitation (*n* = 8, 24.2% vs. *n* = 7, 21.2%) but not significantly different (0.93).

When we compared the frequency of LUS signs separating patients into SSc-ILD and SSc-wILD groups (Table 2), participants with SSc-ILD had more LUS signs for all parameters evaluated. This higher frequency was observed both pre-PGRP and post-PGRP.

### 3.3. Lung Ultrasound—Quantity of Signs

In the analysis of the quantity of lung ultrasound signals (Table 3), before participation in the PGRP, the median presence of B-lines > two was three (0–7), while after the program, it was three (0–6) (*p* = 0.020). Regarding the presence of coalescent B-lines, the values were zero (0–2) before the PGRP and zero (0–2) after (*p* = 0.79). Regarding subpleural consolidations, before the PGRP, the value was zero (0–0.5), compared to zero (0–0) after the program (*p* = 0.032). The sum of the findings in the six areas of each hemithorax evaluated in the LUS, that is, the aeration score, was 8.0 (0–16.5) before the PGRP and 3.0 (0–16) after (*p* = 0.013).

When we compared the impact of a PGRP on the number of LUS signs by separating patients into SSc-ILD and SSc-wILD groups (Table 4), the main change observed was a reduction in the presence of B-lines > two between the pre-PGRP [6 (4–11)] and post-PGRP [5 (2.8–7.3)] moments in the SSc-ILD group, with a statistical significance *p* = 0.0004, which did not occur among patients with SSc-wILD. This change in pattern was reflected in the aeration score. Pre-PGRP, women with SSc-ILD had a higher aeration score than women with SSc-wILD [15.5 (9.5–20.5 vs. 0 (0–2), *p* < 0.0001)]. Post-PGRP, women with SSc-ILD also had higher aeration scores than women with SSc-wILD [13.5 (7.5–19.3 vs. 0 (0–3), *p* = 0.0001)]. When comparing absolute deltas (pre- vs. post-PGRP), there was a decrease in aeration score after RDOF in women with SSc-ILD compared to women with SSc-wILD [−0.50 (−3.3–0) vs. 0 (0–0), *p* = 0.016].

## 4. Discussion

Rehabilitation programs play a critical role in the management of SSc, even delaying its disabling impacts on the respiratory system [14]. Home rehabilitation through a PGRP has become a widely used resource for people with chronic non-communicable diseases and is, therefore, strategic for patients with SSc. This modality can reduce treatment costs, increase the number of patients who can be reached by the program, adapt to the individual characteristics and routine of each patient, strengthen the relationship between physiotherapist and patient, and facilitate health education with the aim of making exercise a habit [11,12,13,15]. To date, no study has focused on the response of the lungs in patients with SSc to a PGRP. The main findings of the present study suggest that LUS was able to detect pre- and post-PGRP changes in patients with SSc-wILD and SSc-ILD and, after 12 weeks of a PGRP, LUS was able to detect an improvement in the aeration score in patients with SSc-ILD. To our knowledge, this is the first study that evaluated a PGRP using LUS in patients with SSc-ILD.

There are several ways in which the lungs can be affected in SSc, with ILD being the most prevalent. These interstitial changes are characterized by a diffuse distribution throughout the lung parenchyma. CT plays a fundamental role in identifying lung lesions in SSc, assessing their location and extent, monitoring disease progression, and guiding treatment [16]. In this study, we chose LUS because of its advantages, such as safety, accessibility, speed, reproducibility, low cost, and good patient acceptance. In addition, this examination can be repeated periodically in a short period of time because it does not involve ionizing radiation and has an agreement rate of over 80% with CT in detecting ILD [17]. Of note, some patients in our study had B-lines on LUS even though there was no diagnosis of ILD on chest CT. In fact, the presence of B-lines is not diagnostic of ILD in SSc, although the number of B-lines on LUS has been shown to be an independent predictor of the onset of ILD in patients without ILD [18].

At the end of this study, the number of B-lines > two was lower when compared to pre-PGRP. B-lines are a sign of increased density of the peripheral lung parenchyma with partial loss of aeration indicative of pulmonary interstitial syndrome [19,20]. This change in structural pattern is reflected in the aeration score [9]. Before the PGRP, women with SSc-ILD had a higher aeration score than women with SSc-wILD. Post-PGRP, women with SSc-ILD also had higher aeration scores than women with SSc-wILD. When comparing the variation in delta (pre- vs. post-PGRP), there was a significant decrease in the aeration score post-PGRP in women with SSc-ILD compared to women with SSc-wILD in our sample, indicating the reversibility of LUS signs with the implemented exercise program.

A study by Galea and colleagues (2024) [21] investigated the potential contribution of the chest wall muscle area to the ventilatory efficiency and exercise capacity in SSc-wILD. They found a statistically significant positive correlation between chest area and maximal workload in cardiopulmonary exercise testing. Thongchote and colleagues [22] found in their study that physical exercises including self-stretching and strengthening can improve chest mobility, respiratory muscle strength, and pulmonary function in COPD patients. Since the diagnosis of ILD is traditionally based on chest CT, FVC, and DLco on PFTs, and considering biomechanics with a restrictive ventilatory pattern are common in SSc-ILD, exercises that increase thoracic expansion and muscle efficiency, with a consequent increase in lung volumes and capacities, may justify a structural improvement in lung aeration verified by LUS.

The strength of this study is that it shows the number of LUS signals before and after a PGRP in women with SSc. However, some limitations should be noted. First, the sample was small, representative of a single center, and composed only by women. However, women have a higher risk of incident SSc than men [23]. Second, we did not compare LUS with CT, although the aim was to avoid radiation. Third, ultrasound is an examiner-dependent examination, that is, the accuracy of the result depends on the experience of the professional performing it; to reduce this bias in our study, each examination was performed by two physicians, who reached a consensus in case of disagreement. Fourth, rehabilitation was not in person and weekly monitoring was performed asynchronously, although the aim was to increase adherence, coverage, and reduce costs using a PGRP. Despite these limitations, our study may serve as a starting point for randomized controlled trials using larger samples and methods to assess the functional impact and health-related quality of life in patients with SSc who undergo a PGRP to corroborate the clinical relevance of these findings.

## 5. Conclusions

Our results showed that LUS may be a complementary tool to detect pre- and post-PGRP changes in patients with SSc-wILD and SSc-ILD, and that B-lines > two are the most prevalent signal. In addition, we found that women with SSc-ILD have worst aeration scores than women with SSc-wILD. Also, a PGRP can change LUS signals in women with SSc, especially in women with an associated ILD. Therefore, we suggest that LUS could be incorporated into routine monitoring of these patients, and that a PGRP should be applied as non-pharmacological management of SSc in addition to medical treatment.

## Figures and Tables

**Table 1:** Characteristics of participants of the study at baseline.

Variables		Values
**Demographic/** **anthropometric data**	Age (years)	48.8 ± 13
	Time since diagnosis (year)	8 (3–15)
	Weight (kg)	72.8 ± 10.7
	Height (m)	1.61 ± 0.06
	BMI (kg/m^2^)	28.1 ± 4.1
**Clinical characteristics**	Limited cutaneous SSc (*n*, %)	2 (6.1%)
	Diffuse cutaneous SSc (*n*, %)	31 (93.9%)
	Interstitial lung disease (*n*, %)	18 (54.5%)
	Pulmonary hypertension (*n*, %)	2 (6.1%)
	Systemic arterial hypertension (*n*, %)	14 (42.4%)
	Diabetes mellitus (*n*, %)	4 (12.1%)
	Dyslipidemia (*n*, %)	3 (9.1%)
	Gastrointestinal symptoms (*n*, %)	6 (18.2%)
	Renal disease (*n*, %)	1 (3%)
**Serology**	Anti-TOPO I positivity (*n*, %)	22 (66.7%)
	Anti-RNAP III positivity (*n*, %)	8 (24.2%)
	Anti-centromere positivity (*n*, %)	3 (9.1%)

Results expressed as the mean ± standard deviation or as the median and interquartile range or number (%). ILD, interstitial lung disease (diagnoses by computed tomography); PH, pulmonary hypertension; anti-TOPO I, antibodies against topoisomerase I; anti-RNAP III, antibodies against RNA polymerase III; anti-Th/To, antibodies to Th/To ribonucleoprotein.

**Table 2:** Frequency of lung ultrasound signals in women with SSc assessed pre- and post-physiotherapy-guided rehabilitation program.

	Pre-PGRP			Post-PGRP		
	SSc with ILD	SSc Without ILD	*p* Value	SSc with ILD	SSc Without ILD	*p* Value
B-lines > two	16 (88.9%)	5 (33.3%)	**<0.0001**	16 (88.9%)	5 (33.3%)	**<0.0001**
Coalescent B-lines	11 (61.1%)	1 (6.7%)	**0.001**	11 (61.1%)	1 (6.7%)	**0.001**
Subpleural consolidations	7 (38.9%)	1 (6.7%)	**0.038**	7 (38.9%)	0 (0%)	**0.007**

PGRP, physiotherapy-guided rehabilitation program; SSc, systemic sclerosis; ILD, interstitial lung disease (diagnoses by computed tomography).

**Table 3:** Lung ultrasound signals in women with SSc assessed pre- and post-physiotherapy-guided rehabilitation program.

Lung Ultrasound Signals	Pre-PGRP	Post-PGRP	*p* Value
B-lines > two	3 (0–7)	3 (0–6)	**0.020**
Coalescent B-lines	0 (0–2)	0 (0–2)	0.79
Subpleural consolidations	0 (0–0.5)	0 (0–0)	0.032
Aeration score (points)	8 (0–16.5)	3 (0–16)	**0.013**

Results expressed as the median (interquartile range). PGRP, physiotherapy-guided rehabilitation program.

**Table 4:** Comparison between lung ultrasound signals in women with systemic sclerosis with and without ILD assessed pre- and post-physiotherapy-guided rehabilitation program.

	Pre-PGRP			Post-PGRP			
	SSc with ILD	SSc Without ILD	*p* Value	SSc with ILD	SSc Without ILD	*p* Value	*p* Value *
B-lines > two	6 (4–11)	0 (0–2)	**<0.0001**	5 (2.8–7.3)	0 (0–1)	**0.0004**	**0.002**
Coalescent B-lines	2 (0–5.3)	0 (0–2)	**0.0009**	2 (0–5)	0 (0–0)	**0.0009**	0.59
Subpleural consolidations	0 (0–2)	0 (0–0)	0.026	0 (0–2)	0 (0–0)	0.008	0.27
Aeration score (points)	15.5 (9.5–20.5)	0 (0–16)	**<0.0001**	13.5 (−3.3–0)	0 (0–3)	**<0.0001**	**0.016**

Results expressed as the median (interquartile range). PGRP, physiotherapy-guided rehabilitation program. ILD, interstitial lung disease. * Refers to the difference between the absolute deltas obtained by subtracting post-PGRP values from pre-PGRP values between the SSc with ILD and SSc without ILD groups.

## Data Availability

The data supporting the conclusions of this article can be made available by the authors upon reasonable request.

## Abbreviations

The following abbreviations are used in this manuscript:

ADL	Activities of daily living
CT	Computed tomography
DLco	Diffusing capacity of the lungs for carbon monoxide
EULAR	European Alliance of Associations for Rheumatology
FVC	Forced vital capacity
HRQoL	Health-related quality of life
ILD	Interstitial lung disease
LUS	Lung ultrasound
PFTs	Pulmonary function tests
PGRP	Physiotherapy-guided rehabilitation program
SSc	Systemic sclerosis
SSc-ILD	Systemic sclerosis with interstitial lung disease
SSc-wILD	Systemic sclerosis without interstitial lung disease
UIP	Usual interstitial pneumonia

## References

[1] Volkmann E.R., Andréasson K., Smith V. (2023). Systemic sclerosis. Lancet.

[2] Van Den Hoogen F., Khanna D., Fransen J., Johnson S.R., Baron M., Tyndall A., Matucci-Cerinic M., Naden P., Medsger T.A., Careira P.E. (2013). 2013 classification criteria for systemic sclerosis: An american college of rheumatology/European league against rheumatism collaborative initiative. Arthritis Rheum..

[3] Kayser C., de Oliveira Delgado S.M., Zimmermann A.F., Horimoto A.M.C., Del Rio A.P.T., de Souza Müller C., Camargo C.Z., Lupo C.M., de Moraes D.A., Do Rosário E Souza E.J. (2024). 2023 Brazilian Society of Rheumatology guidelines for the treatment of systemic sclerosis. Adv. Rheumatol..

[4] Bergamasco A., Hartmann N., Wallace L., Verpillat P. (2019). Epidemiology of systemic sclerosis and systemic sclerosis-associated interstitial lung disease. Clin. Epidemiol..

[5] Mohammad Reza Beigi D., Pellegrino G., Loconte M., Landini N., Mattone M., Paone G., Truglia S., Di Ciommo F.R., Bisconti I., Cadar M. (2024). Lung ultrasound compared to computed tomography detection and automated quantification of systemic sclerosis-Associated interstitial lung disease: Preliminary study. Rheumatology.

[6] Godfrey S., Bluestone R., Higgs B.E. (1969). Lung function and the response to exercise in systemic sclerosis. Thorax.

[7] Parodis I., Girard-Guyonvarc’H C., Arnaud L., Distler O., Domján A., Van Den Ende C.H.M., Fligelstone K., Kocher A., Larosa M., Lau M. (2024). EULAR recommendations for the non-pharmacological management of systemic lupus erythematosus and systemic sclerosis. Ann. Rheum. Dis..

[8] Poerio A., Carlicchi E., Zompatori M. (2023). Diagnosis of interstitial lung disease (ILD) secondary to systemic sclerosis (SSc) and rheumatoid arthritis (RA) and identification of ‘progressive pulmonary fibrosis’ using chest CT: A narrative review. Clin. Exp. Med..

[9] Reyes-Long S., Gutierrez M., Clavijo-Cornejo D., Alfaro-Rodríguez A., González-Sámano K., Luis Cortes-Altamirano J., Muñoz-Louis R., Cruz-Arenas E., Camargo K., Gonzalez F. (2021). Subclinical interstitial lung disease in patients with systemic sclerosis. A pilot study on the role of ultrasound. Reumatol. Clin..

[10] de Alegria S.G., Litrento P.F., Farias I.O., Mafort T.T., Lopes A.J. (2022). Can home rehabilitation impact impulse oscillometry and lung ultrasound findings in patients with scleroderma-associated interstitial lung disease? A pilot study. BMC Res. Notes.

[11] de Alegria S.G., Azevedo B.L.P.A., Oliveira J.G.M., da Silva M.M., Gardel D.G., Mafort T.T., José Lopes A. (2023). Home-based rehabilitation improves functional capacity and quality of life in women with systemic sclerosis: A preliminary study. J. Back Musculoskelet. Rehabil..

[12] Almeida C.H.S., Reis L.F.F., Nascimento L.P.A.S., Soares A.R., Maioli M.C.P., Lopes A.J. (2021). Therapist-oriented home rehabilitation for adults with sickle cell anemia: Effects on muscle strength, functional capacity, and quality of life. Hematology.

[13] Lima T.R.L., Kasuki L., Gadelha M., Lopes A.J. (2019). Physical exercise improves functional capacity and quality of life in patients with acromegaly: A 12-week follow-up study. Endocrine.

[14] Murphy S.L., Poole J.L., Chen Y.T., Lescoat A., Khanna D. (2022). Rehabilitation interventions in systemic sclerosis: A systematic review and future directions. Arthritis Care Res..

[15] Civi Karaaslan T., Tarakci E., Keles O., Aslan Keles Y., Ugurlu S. (2023). Comparison of telerehabilitation methods for patients with systemic sclerosis in the COVID-19 Era: A randomized controlled study. J. Hand Ther..

[16] Bastos A.L., Corrêa R.A., Ferreira G.A. (2016). Tomography patterns of lung disease in systemic sclerosis. Radiol. Bras..

[17] Gasperine M.L., Gigante A., Iacolare A., Pellicano C., Lucci S., Rosato E. (2020). The predictive role of lung ultrasound in progression of scleroderma interstitial lung disease. Clin. Rheumatol..

[18] Gargani L., Bruni C., Romei C., Frumento P., Moreo A., Agoston G., Guiducci S., Bellando-Randone S., Lepri G., Belloli L. (2020). Prognostic value of lung ultrasound B-Lines in systemic sclerosis. Chest.

[19] Volpicelli G. (2020). Lung ultrasound B-lines in interstitial lung disease: Moving from diagnosis to prognostic stratification. Chest.

[20] Vicente-Rabaneda E.F., Bong D.A., Castañeda S., Möller I. (2021). Use of ultrasound to diagnose and monitor interstitial lung disease in rheumatic diseases. Clin. Rheumatol..

[21] Galea N., Colalillo A., Paciulli S., Pellicano C., Giannetti M., Possente E. (2025). Chest wall muscle area, ventilatory efficiency and exercise capacity in systemic sclerosis. Intern. Emerg. Med..

[22] Thongchote K., Chinwaro U., Lapmanee S. (2024). Effects of scapulothoracic exercises on chest mobility, respiratory muscle strength, and pulmonary function in male COPD patients with forward shoulder posture: A randomized controlled trial. F1000Res.

[23] Kwon O.C., Han K., Park M.C. (2025). Sex differences in the risk of incident systemic sclerosis: A nationwide population-based study with subgroup analyses. Sci. Rep..

